# Congenital intestinal malrotation, duodenal obstruction combined with dextrocardia: a rare case report

**DOI:** 10.3389/fped.2025.1554891

**Published:** 2025-04-08

**Authors:** Jianhong Yan, Hang Yang, Han Xiao, Chuanxin Li

**Affiliations:** Department of General Surgery, Kunming Children’s Hospital, Kunming, China

**Keywords:** intestinal malrotation, duodenal obstruction, dextrocardia, premature infant, Ladd's procedure

## Abstract

**Background:**

Both intestinal malrotation and situs inversus are usually asymptomatic and extremely rare. We reported a case of congenital intestinal malrotation, duodenal obstruction, dextrocardia, and pancreatic and splenic hypoplasia in a newborn.

**Case presentation:**

The patient was a 17-day-old premature infant who had experienced recurrent vomiting for 5 days. Upper gastrointestinal and small intestine contrast imaging indicated intestinal malrotation and duodenal obstruction. Abdominal contrast-enhanced CT showed a small, underdeveloped pancreas, multiple nodular soft tissue densities in the area anterior to the left kidney and posterior to the stomach, and unclear splenic venous and arterial structures. Cardiac CT revealed dextrocardia with an atrial septal defect (secundum type). The laparoscopic Ladd's procedure was performed. The postoperative course was uneventful, and the patient recovered well during a 4-month follow-up.

**Conclusions:**

This patient was very young and presented with multiple abnormalities. This case highlights the importance of early diagnosis, timely referral, and management of such rare congenital anomalies to achieve favorable outcomes.

## 1 Introduction

Intestinal malrotation is a congenital anomaly characterized by insufficient or incomplete rotation of the fetal intestines around the mesenteric arter*y* axis during development (1). Its incidence in the literature is generally reported to be 0.2% (2–4), however, the actual incidence remains unknown as some rotational abnormalities remain asymptomatic for the whole lifetime. Occasionally, these patients would present duodenal obstruction, which is the most common site of neonatal intestinal obstruction, accounting for 50% of all cases of intestinal atresia (5). In clinical practice, the diagnosis of intestinal malrotation is mainly relied on upper gastrointestinal contrast imaging and B-scan ultrasonography, which could identify specific abnormalities indicative of malrotation (6, 7).

Situs inversus is a rare condition characterized by a mirror-image positioning of the thoracic and abdominal organs, which it can be classified into complete and partial types (8). Dextrocardia is a form of partial situs inversus, in which the majority or entire heart is located on the right side of the chest at birth, with an incidence of approximately 0.01% among live-born infants (9). Patients with situs anomalies often present with various gastrointestinal abnormalities, including right-sided stomach, midline liver, biliary anomalies, intestinal malrotation, and multiple or dysfunctional spleens (10). These organ positioning abnormalities often coincide with other developmental irregularities across various systems, such as congenital pancreatic and splenic hypoplasia, congenital heart disease and vascular dysplasia (11, 12).

In this study, we reported an extremely rare case presenting congenital intestinal malrotation, duodenal obstruction, dextrocardia, absent inferior vena cava (IVC) and pancreatic and splenic hypoplasia simultaneously, and reported our experiences on the diagnosis and treatment of such condition.

## 2 Case presentation

A 17-day-old female infant was admitted to the pediatric department in a local hospital due to frequent vomiting for 5 days (at least 3 times per day) without an obvious cause. Her mother received ultrasonography at a gestational age of 25 weeks, which revealed abnormal fetal heart position, inverted atrium, left ventricular loop (mostly considered mirror dextrocardia), unclear display of the inferior vena cava, widened umbilical vein parallel to the descending aorta, disconnection of the inferior vena cava, and persistent right umbilical vein. She was delivered at a gestational age of 35 weeks and 6 days with a birth weight of 2.5 kg. Upon admission, the infant was treated with nasal continuous positive airway pressure (NCPAP), and high-flow oxygen therapy. The combination of ampicillin sodium and cefoperazone was utilized for treating infection. Phototherapy was given as the patient showed jaundice. The other treatment included intravenous nutritional support, and atropine for maintaining a normal heart rate. This study was approved by Kunming Children's Hospital. The studies were conducted in accordance with the local legislation and institutional requirements. Written informed consent for participation in this study was provided by the participants' legal guardians.

For the vomiting, merely bile gastric contents were noticed. There was no abdominal distension, diarrhea, or fever, and the vomiting was attenuated after 1 day of fasting. However, after feeding on formula milk (25 ml–50 ml once, with a time interval of 2–3 h), the infant showed frequent vomiting again (about 7 times per day), with the vomit consisting of undigested formula milk in a yellow and green color.

**Figure 1:**
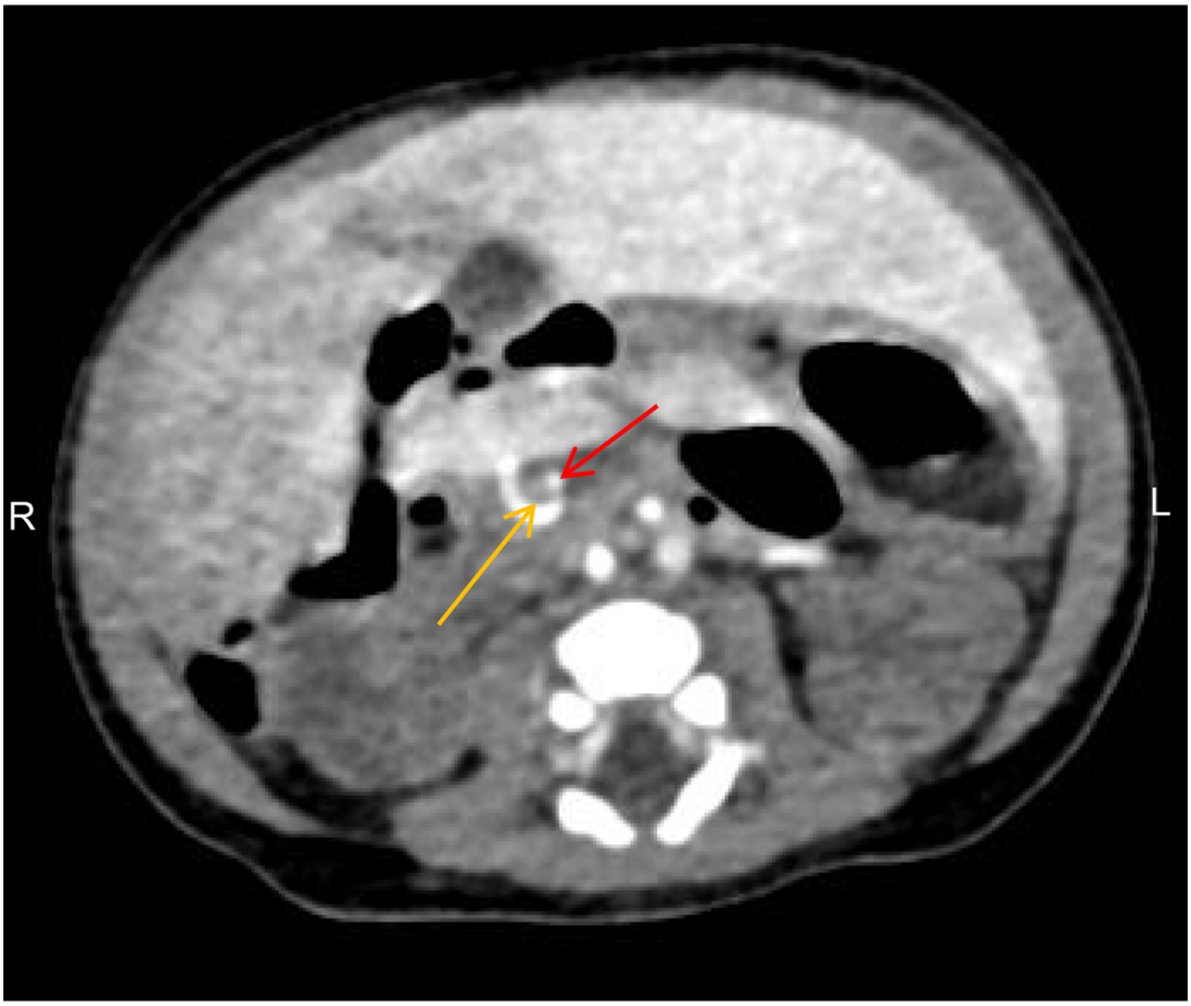
Abdominal CT showing the superior mesenteric vein (yellow arrow) located to the left of the superior mesenteric artery (red arrow), with the “whirlpool sign” visible, indicative of intestinal malrotation.

**Figure 2:**
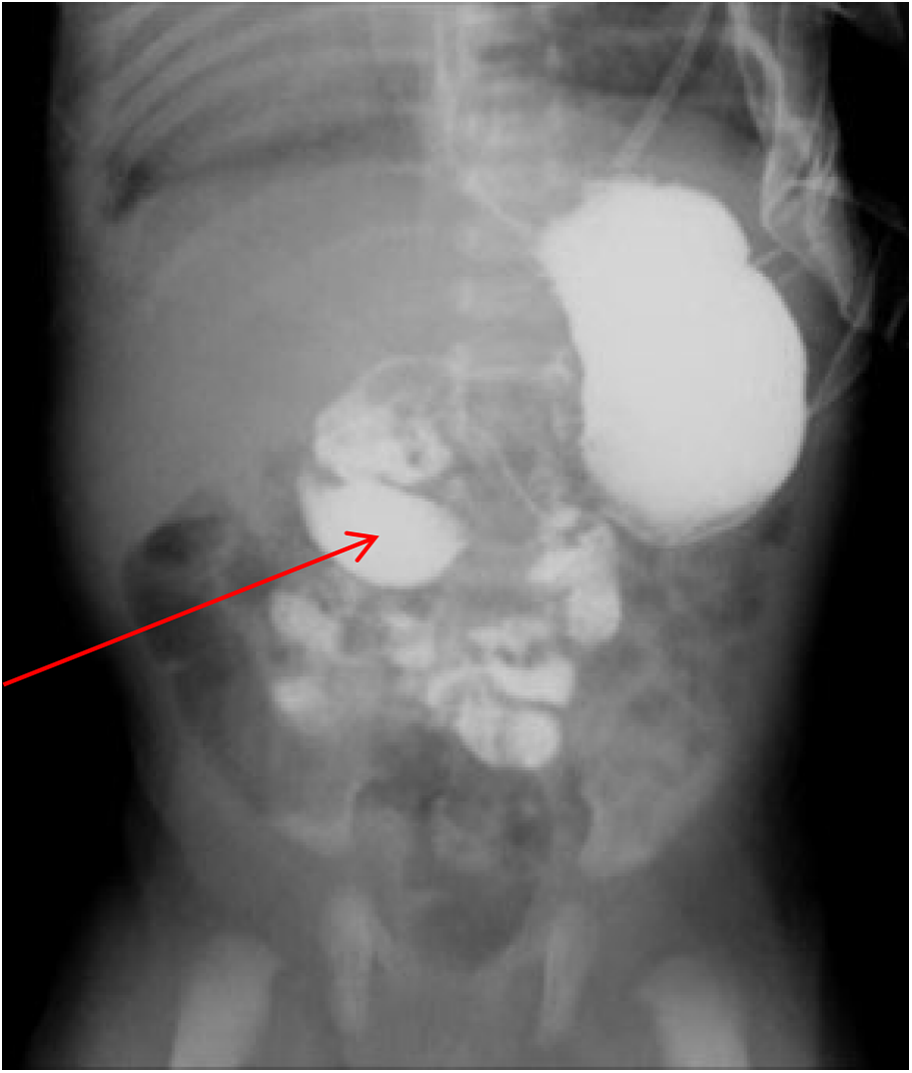
Small intestine contrast imaging. The imaging showed that the child had congenital intestinal malrotation and caused duodenal obstruction (as indicated by arrows).

**Figure 3:**
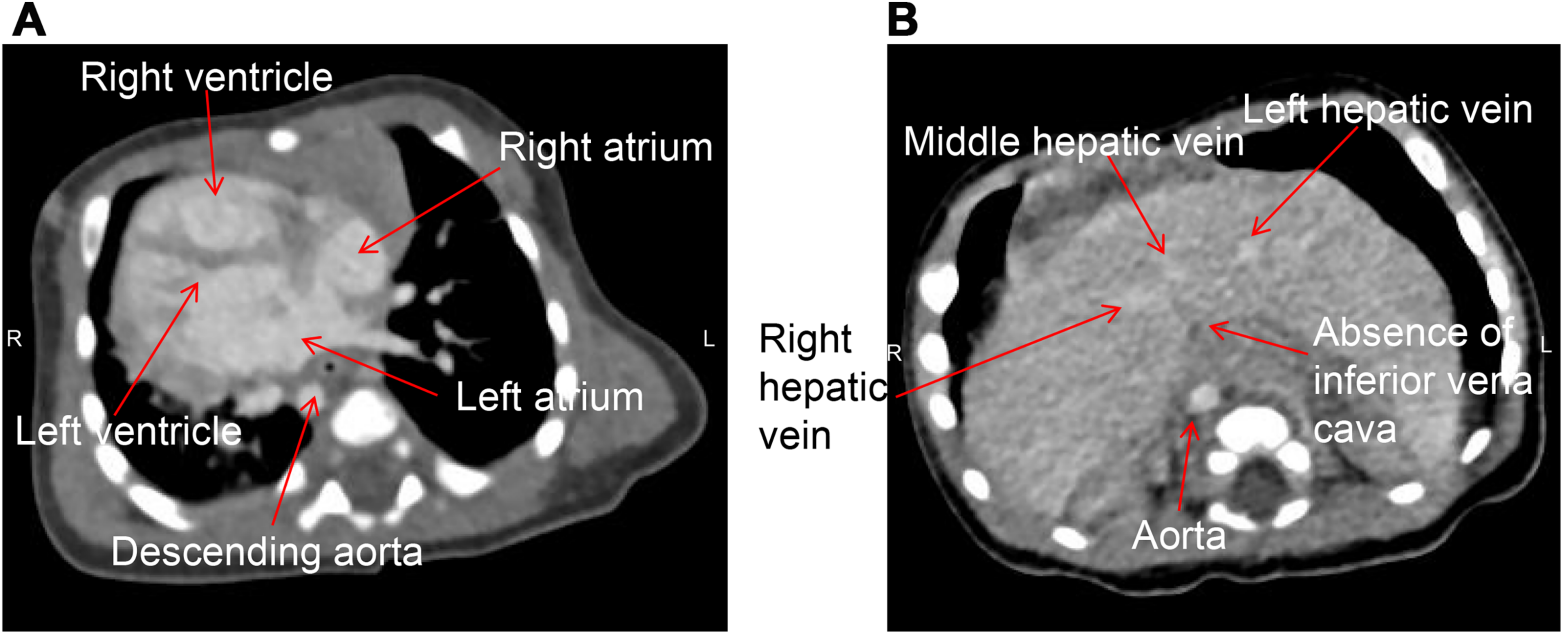
Cardiac CT of the patient taken in the supine position. **(A)** Dextrocardia with the apex pointing to the right, right-sided aortic arch, and descending along the right side of the spine; **(B)** the absence of the hepatic segment of the IVC, with the hepatic vein draining directly into the right atrium.

**Figure 4:**
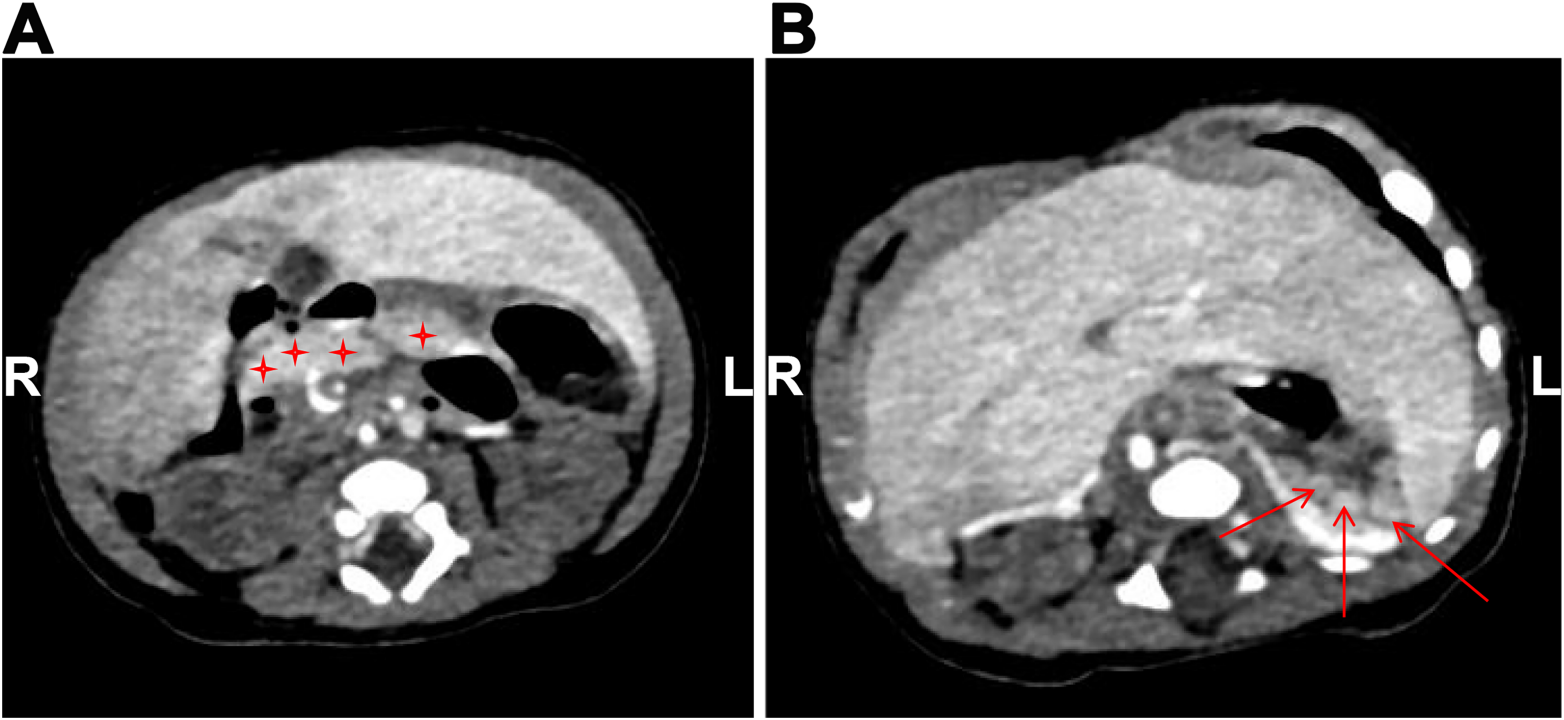
Abdominal contrast-enhanced CT image. **(A)** The pancreas is small in size, suggesting hypoplasia (as indicated by asterisks); **(B)** multiple nodular soft tissue densities in the area anterior to the left kidney and posterior to the stomach, indicating splenic hypoplasia (as indicated by arrows).

The infant was then transferred to our department for further treatment. Upon admission, the patient showed no fever or convulsions, but poor mental response. Prior to surgery, the patient received conventional imaging examinations and routine blood test. Blood tests revealed an elevated platelet count (5.72 × 10^11^/L). Abdominal CT and small intestine contrast imaging indicated intestinal malrotation (Figure 1) and duodenal obstruction (Figure 2). Cardiac CT showed dextrocardia, atrial septal defect (Figure 3A); the IVC in the hepatic segment was not visualized, and the hepatic veins directly entered the right atrium, suggesting the absence of the hepatic segment of the IVC (Figure 3B). Abdominal contrast-enhanced CT revealed hypoplasia of the pancreas (Figure 4A); multiple nodular soft tissue densities were noted in the area anterior to the left kidney and behind the stomach, while the splenic vessels were unclear, suggesting splenic hypoplasia (Figure 4B).

The Ladd's procedure was utilized for the treatment after general anesthesia. Specifically, the patient was in a supine position. A 5 mm cannula was inserted below the umbilicus to establish pneumoperitoneum (pressure: 6 mmHg). Then a laparoscope was introduced, under direct laparoscope vision, a 5 mm cannula was placed in the right upper abdomen, a 3 mm cannula was placed adjacent to the right umbilicus, and the operating instruments were inserted. Exploration of the abdominal cavity revealed that the ileocecal region was located in the upper and middle abdomen, and the midgut twisted 540° clockwise. The transverse colon in the spleen area was found, and the transverse colon was lifted up alternately in a retrograde manner. The twisted intestinal tube was twisted and reset in a counterclockwise direction using it as a reference until the appendix was found and the colon was reset. The cecum and small intestine were lifted up alternately, and the small intestine was reset in a counterclockwise direction. The small intestine and mesentery were found to be intact and fan-shaped. The ascending colon was lifted up, and the electrocoagulation hook was used to fully loosen the Ladd bands and the right gastrocolic ligament connected to the colon until the middle colic vein, and the colon was turned to the left side of the abdominal cavity. The fibrous tissue around the duodenum was loosened until the duodenum became an intra-abdominal organ, and the initial section of the duodenum-jejunum was completely straightened and placed on the right side of the spine. The contracted mesentery was loosened along the jejunum until the ileocecal region. The stomach was inflated through a gastric tube, and the stomach and duodenum were squeezed alternately. The jejunum was quickly inflated, confirming that the loosening was sufficient and the digestive tract was unobstructed. The appendix was routinely removed and the tissue was analyzed pathologically. It was confirmed again that the small intestine was placed on the right side of the abdominal cavity, the ileocecal region was placed in the left upper abdomen, and the colon was placed in the left abdomen. The pneumoperitoneum was released, and the trocar was removed. Finally, the incisions were closed with interrupted sutures using 4-0 absorbable sutures. In the 4-month follow-up, the child was in good health with an obvious increase in the body weight, while no vomiting or adverse events were noticed.

## 3 Discussion

As intestinal malrotation is usually asymptomatic, its diagnosis is still difficult without utilizing imaging techniques (13). Usually, dextrocardia is diagnosed incidentally as it is also asymptomatic. These two conditions are extremely rare in an individual, but they may lead to series of maldevelopments such as duodenal obstruction, as well as other organ involvement. In this study, we reported an extremely rare case showing intestinal malrotation combined with duodenal obstruction, dextrocardia, absence of the IVC, and pancreatic and splenic hypoplasia. We aimed to highlight the rarity of this disorder and share our experience in diagnosis and treatment.

During embryonic development, the intestines reach their normal position and attach within the peritoneal cavity through a process that begins at the fifth week of gestation and continues after birth (14). Abnormal growth can cause the intestinal protruding through the umbilical cord, returning around the 10th–11th week and becoming fixed to the posterior abdominal wall, leading to midgut rotation (14). Acute clinical manifestations of malrotation include peritonitis, small bowel obstruction, or signs of appendicitis (15), some of which were observed in our case. It can also cause dislocation of the appendix from its normal position, resulting in signs that may not be localized to the right lower abdomen (16). Congenital duodenal obstruction may be associated with extrinsic narrowing of the duodenum caused by peritoneal bands, related to intestinal malrotation, or linked to certain syndromes such as VACTERL (17).

Dextrocardia is rare, and its diagnosis can be challenging at any age—whether in the fetus, infant, or child (18). It is typically associated with significant cardiac and cardiovascular malformations, such as atrial septal defects, ventricular septal defects, and transposition of the great arteries (18). Patients with situs anomalies often exhibit multiple gastrointestinal abnormalities. Some studies have shown that situs inversus is associated with intestinal malrotation and neonatal intestinal obstruction, as seen in our case (19). This suggests that dextrocardia is the primary condition in our case, potentially explaining most of the symptoms observed. IVC hypoplasia is one of the rarest IVC anomalies, with a prevalence of 0.0005%–1% in the general population (20). It has been suggested that an abnormal hepatic vein may represent a persistent umbilical mesenteric vein (21). The underlying cause of absent IVC is not fully understood, but it is hypothesized that embryonic developmental defects and in-utero or perinatal thrombosis may play a role (22, 23). The absence of the superior vena cava (SVC) is often associated with cardiac abnormalities, while the absence of the IVC is often associated with abnormalities such as the spleen (24, 25). Our patient also exhibited pancreatic and splenic hypoplasia, possibly due to the absence of the IVC. The coexistence of intestinal malrotation, duodenal obstruction, dextrocardia, absence of the IVC, and pancreatic and splenic hypoplasia in this case is exceedingly rare and has been sparsely reported in the literature.

Situs inversus is usually accompanied by cardiovascular and gastrointestinal malformations, which are also frequently seen in previous case reports. For instance, Zhou et al. reported a case of an annular pancreas combined with intestinal malrotation and duodenal duplication in an 11-year-old man who developed gastroesophageal reflux disease after Ladd's procedure and eventually recovered with duodenostomy (1). Many case reports confirm that there is still a risk of recurrence after Ladd's procedure. Ben et al. reported a partial 18-day-old patient with partial situs inversus, annular pancreas, duodenal obstruction and intestinal malformation, who underwent duodenotomy, diaphragm resection and Ladd's procedure for the treatment. However, he showed intestinal adhesions and consequent intestinal obstruction (26). Seltz et al. reported a case of a 2-week-old neonate with ectopic spleen and intestinal malrotation, followed by duodenal obstruction due to midgut volvulus, who was treated with peritoneal drainage, Ladd's procedure and nutritional support (27). The above cases were similar to our case, all of whom have intestinal malformations caused by situs inversus, along with a risk of postoperative recurrence. The difference was that the patient in this study was younger and presented dextrocardia, which resulted in surgical challenge and a risk of recurrence. Fortunately, our case showed no recurrence in the 4 months follow-up.

The surgical treatment of intestinal malrotation includes detorsion, widening of the mesenteric base, and, when appropriate, division of the Ladd's band. An appendectomy is also commonly performed during the procedure (28). Laparoscopic surgery for the treatment of intestinal malrotation was first reported in 1995 and has become increasingly popular in recent years (29). The potential benefits of laparoscopic surgery include better visualization, smaller incisions, less postoperative pain, shorter hospital stays, superior cosmetic results, and a reduced incidence of adhesive bowel obstruction and wound infections (30, 31). However, due to the small size and limited workspace and visibility in neonates, previous studies recommended against the use of laparoscopic approaches in this age group (32). In contrast, other studies have reported successful laparoscopic surgeries in neonates as young as 3 days old, demonstrating that an experienced surgical team can safely perform laparoscopic procedures in neonates using instruments with a diameter of 3 mm (28, 33). Furthermore, if the expected outcome cannot be achieved via laparoscopy, preparation should be made to convert to an open surgical approach (34).

The challenges in performing laparoscopic Ladd's procedure in our case stem from the fact that the patient is a premature infant (35 weeks and 6 days gestational age), extremely young (17 days old), and the case is rare, with underdeveloped organs and associated dextrocardia. There is also the potential for progressive deterioration, which could lead to multiple organ failure. During surgery, there was a possibility that the severity of the malrotation could preclude the procedure, necessitating a change in the surgical approach (such as bowel resection, anastomosis, or enterostomy). Postoperatively, aggressive infection control and symptomatic support were required. Despite successful surgery, there remains the possibility of complications such as intestinal adhesions, bowel obstruction, intestinal perforation, fistulas, or short bowel syndrome, which may necessitate further surgical interventions.

The main postoperative complications of laparoscopic Ladd's procedure are adhesive intestinal obstruction and midgut volvulus. Recurrent volvulus rarely occurs, but adhesive intestinal obstruction is more common (35, 36). Malrotation is a recognized risk factor for adhesion-related obstruction in neonates (37). In a retrospective study involving 161 patients who underwent Ladd's procedure with an average follow-up of 5 years. 8.7% of patients showed complications after surgery and 5.6% of patients had adhesive small bowel obstruction (38). To prevent adhesive intestinal obstruction after Ladd's procedure, it is important to avoid intestinal ischemia and adhesions to the wide exposed area of the mesentery by expanding the mesenteric base (35). Another major problem with laparoscopic treatment of malrotation is the risk of recurrent midgut volvulus. Catania et al. reported a 3.5% recurrence rate for midgut rotation, primarily due to inadequate visualization and inability to reduce midgut volvulus (30). The original report by Van der Zee et al. emphasized that at the end of the surgical dissection, the surgeon must be able to easily follow the straightened duodenum and the entire small intestine with a large widened mesenteric floor, a key point that may reduce the risk of recurrence due to inadequate surgery (29). Abdominal surgery is the most reliable and commonly used procedure for reoperation after the Ladd's procedure. In the study by Zhu et al., all reoperations were performed by open laparotomy, and there were no postoperative complications and good outcomes after reoperation (39). In conclusion, parents of these children must be carefully educated about the possibility of small-bowel obstruction and midgut volvulus.

The limitations of this report are also evident. Given the relatively short duration since the surgical treatment, there is limited prognostic data, and effective results from follow-up are lacking. Since this case involves a premature infant with multiple organ underdevelopment, we will continue long-term observation in the future to provide more valuable clinical insights for the diagnosis, treatment, and management of similar cases.

## 4 Conclusion

The simultaneous occurrence of intestinal malrotation, duodenal obstruction, dextrocardia, absence of the IVC, and pancreatic and splenic hypoplasia is extremely rare. Preoperative imaging, thorough gastrointestinal evaluation, and meticulous surgical technique are essential to avoid misdiagnosis, missed diagnoses, or the need for repeat surgeries.

## Data Availability

The original contributions presented in the study are included in the article/Supplementary Material, further inquiries can be directed to the corresponding author.
